# Regular Patterns for Proteome-Wide Distribution of Protein Abundance across Species

**DOI:** 10.1371/journal.pone.0032423

**Published:** 2012-03-09

**Authors:** Fan Zhong, Dong Yang, Yunwei Hao, Chengzhao Lin, Ying Jiang, Wantao Ying, Songfeng Wu, Yunping Zhu, Siqi Liu, Pengyuan Yang, Xiaohong Qian, Fuchu He

**Affiliations:** 1 State Key Laboratory of Proteomics, Beijing Proteome Research Center, Beijing Institute of Radiation Medicine, Beijing, China; 2 Institutes of Biomedical Sciences, Department of Chemistry, Fudan University, Shanghai, China; 3 National Engineering Research Center for Protein Drugs, Beijing, China; 4 Beijing Genomics Institute, Chinese Academy of Sciences, Beijing, China; University of South Florida, United States of America

## Abstract

A proteome of the bio-entity, including cell, tissue, organ, and organism, consists of proteins of diverse abundance. The principle that determines the abundance of different proteins in a proteome is of fundamental significance for an understanding of the building blocks of the bio-entity. Here, we report three regular patterns in the proteome-wide distribution of protein abundance across species such as human, mouse, fly, worm, yeast, and bacteria: in most cases, protein abundance is positively correlated with the protein's origination time or sequence conservation during evolution; it is negatively correlated with the protein's domain number and positively correlated with domain coverage in protein structure, and the correlations became stronger during the course of evolution; protein abundance can be further stratified by the function of the protein, whereby proteins that act on material conversion and transportation (mass category) are more abundant than those that act on information modulation (information category). Thus, protein abundance is intrinsically related to the protein's inherent characters of evolution, structure, and function.

## Introduction

As the endpoint of the central dogma of biology, proteins are central players of most biological processes, and their abundances are regulated precisely to meet with dynamic functional requirement [1]. As the result of regulatory and evolutionary processes, a proteome may span 6 to 12 orders of magnitude among abundance distribution [2]. Exploring the principle controlling protein abundance is of great importance for the deep understanding of the building blocks of a bio-entity. Previous studies have made great progress about this open question [3]. In general, genes with higher mRNA/protein expression level tend to evolve slowly [4]–[6], have less intronic DNA [7], code for smaller proteins [8], [9], have higher biases of amino acid composition [10], [11] and codon usage [11]–[13], and tend to carry out “core functions” [11], [14]. However, these analyses are mostly based on high throughput transcript expression data, or proteome data only from single species. What' more, previous studies didn't pay attentions to the relationship between protein abundance and its character of domain composition. There is lack of systematic analysis to explore the relationship between protein abundance and protein's inherent characters at whole proteome level across species.

To this end, it is necessary to identify and quantify proteins at proteome-wide. With the fast advancement of quantitative proteomics based on mass spectrometry, protein abundance can be measured at the whole proteome level, making biological rule discovery possible. The Human Liver Proteome Project (HLPP) [15], orchestrated by the Human Proteome Organization (HUPO), is the first global survey of proteomes for a human organ, and has produced a comprehensive and reliable dataset [16]. Moreover, some quantitative proteome datasets have been generated from other species, such as *M. musculus* [17], [18], *D. melanogaster* [19], *C. elegans* [20], *S. cerevisiae* [21], and *E. coli* [11]. We analysed these data and uncovered three regular patterns regarding the proteome-wide distribution of protein abundance in the context of evolution, structure, and function.

## Materials and Methods

### 1. Protein quantification

The quantification of protein abundance in the human liver was estimated using the spectral counts (SC) method. The spectral count index (*SCI*), an adjusted *SC* in accounting for the theoretic tryptic-peptide number of protein, was calculated following the reported methods [16]. To integrate data from multiple data sources, various *SCI* values from different experimental batches were normalized, and this normalized *SCI* value was termed the spectral count index normalized (*SCIN*) [16]. The quantitative proteomes of the mouse liver [17], *D. melanogaster* [19], and *C. elegans* [20] were also calculated according to the SCI method. Protein abundance in the mouse renal cortex [18], *S. cerevisiae* [21], and *E. coli* [11] proteome datasets were presented by their normalized spectral abundance factor (*NSAF*) value and copies per cell value from original papers, respectively.

### 2. Functional and subcellular categorization by Gene Ontology

All the gene products of seven datasets (proteomes of human liver, mouse liver, mouse kidney, *D. melanogaster*, *C. elegans*, and *E. coli*) were categorized into given function and subcellular classes by Gene Ontology (GO) annotations [22] according to the Generic GO Slim v1.770 standard.

To obtain some specialized functional categories such as “meiosis” and “cytokinesis”, functional and subcellular categories of yeast gene products were assigned automatically by the GO Slim Mapper system in the *Saccharomyces* Genome Database (SGD) according to the “Yeast GO Slim” standard.

### 3. Estimation of protein origin time and sequence conservation

Protein origin time was estimated by species diversity time [23]. Proteins of the seven proteome datasets were assigned orthology group IDs by OrthoMCL v2. The origin time of an orthology group was estimated by the latest diversity time that can cover all the species involved. All the 87 species in OrthoMCL were used to demarcate the orthology group origin time. The diversity time tree of all the 87 species in OrthoMCL was constructed (**Fig. S1A**), and then was simplified to 7 significant diversity time nodes (**Fig. S1B**). The protein sequences of the seven proteome datasets were extracted, and sequence conservation was determined using the bi-BLAST method with BioEdit software.

### 4. Functional enrichment analysis

The analysis was carried out using the web-accessible DAVID (Database for Annotation, Visualization and Integrated Discovery) tool [24]. DAVID can recognize the IPI identifier from the human liver and the mouse liver datasets, UniProt AC from the mouse kidney, the *D. melanogaster* and the *E. coli* datasets, WBGene ID (a type of ID in WormBase) from the *C. elegans* dataset, and the SGD (Saccharomyces Genome Database) ID from the *S. cerevisiae* dataset. Medium classification stringency and 12 items were chosen for enrichment calculation: COG_ONTOLOGY, SP_PIR_KEYWORDS, UP_SEQ_FEATURE, GOTERM_BP_FAT, GOTERM_MF_FAT, KEGG_PATHWAY, BIOCARTA, BBID, INTERPRO, PIR_SUPERFAMILY, SMART, and OMIM_DISEASE. To avoid high enrichment score disturbance of cellular component categories, GOTERM_CC_FAT was neglected on purpose.

### 5. Definition of protein domain characteristic parameters

Protein domains were defined essentially as described previously [25], using RPS-BLAST against the Pfam [26] and SMART [27] databases with the *E*-value 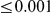 after masking low-complexity regions. The domain number (*DN*) in one protein was calculated including the domain repeats in one protein. Domain coverage (*DC*) refers the percentage of the entire length of a protein occupied by domains. *DC* was divided by its corresponding *DN* to get the *DC*/*DN* value. Protein-protein interaction (PPI) domains were defined through the InterDom database [28]. PPI domain number (*PPI_DN*) refers the number of PPI domains in one protein, and PPI domain coverage (*PPI_DC*) means the percentage of the entire length of a protein occupied by PPI domains. *PPI_DC*/*PPI_DN* was calculated by dividing *PPI_DC* by *PPI_DN*.

### 6. Statistical analysis

All difference-tests in our analysis were carried out by using the Wilcoxon rank sum test, a non-parametric statistical hypothesis test for assessing whether two independent samples of observations have equally large values. All the correlations were defined on the non-parametric Spearman rank correlation, which assesses how well the relationship between two variables can be described using a monotonic function. We perform Wilcoxon rank sum test and spearman rank correlation using MATLAB 7.11.0. Predictive power means the probability that any two random pairs of proteins meet with the general trend. The predictive power value of each correlation was calculated based on pairs of randomly sampled proteins in each proteome dataset, and is represented by the percentage of the true predictions over all the protein pairs selected. We selected various subsets of protein pairs with a minimum rank difference percentage from 0 to 95%, and obtained the minimum and maximum predictive power values for each correlation [29].

## Results

### 1. Evolutionary rule in protein abundance: origination and conservation versus abundance

**Figure 1:**
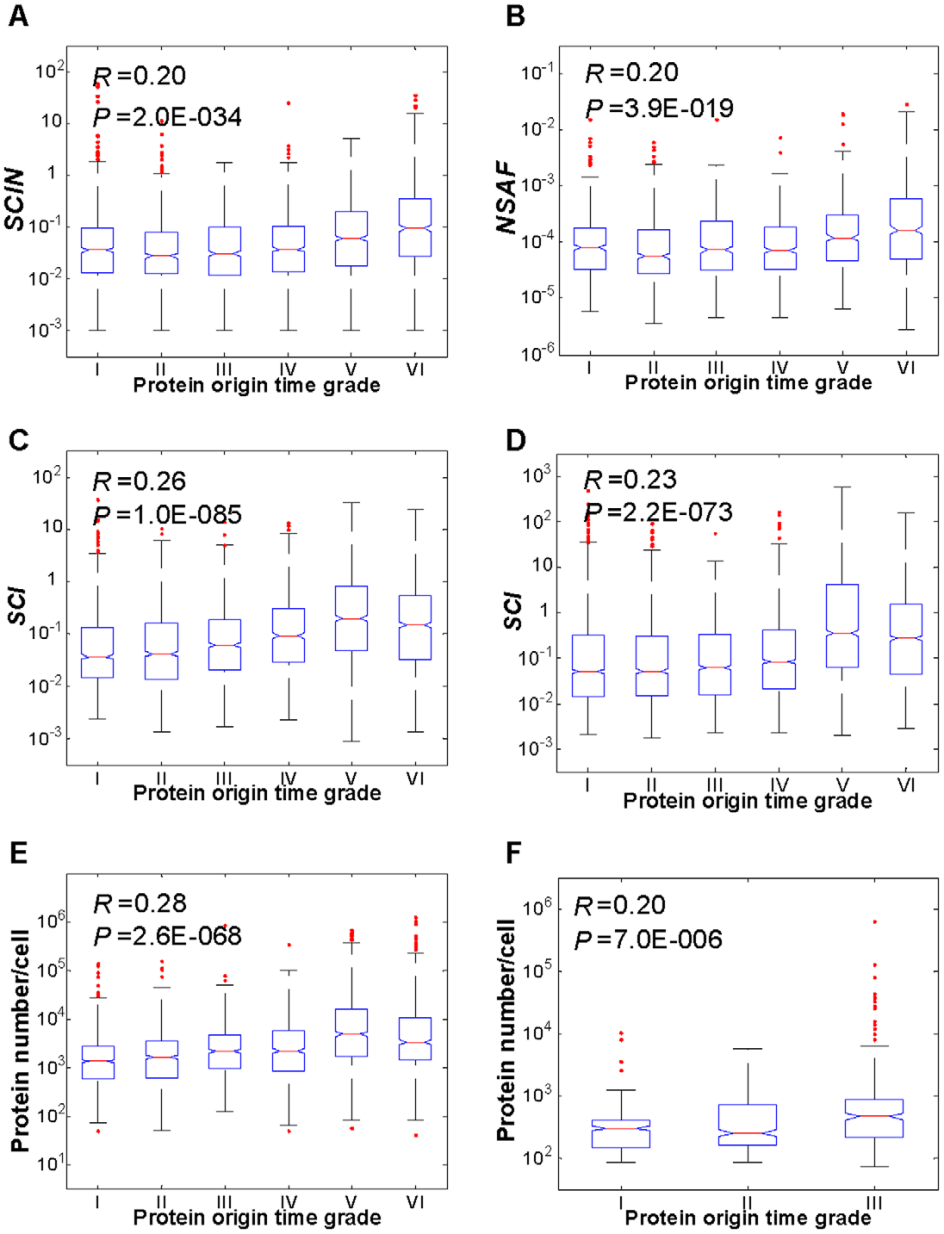
Proteome-wide correlation of proteins' abundance with their origin time across six species. The relationship between origin time and abundance of proteins in *H. sapiens* (**A**), *M. musculus* (**B**), *D. melanogaster* (**C**), *C. elegans* (**D**), *S. cerevisiae* (**E**), and *E. coli* (**F**) were analyzed by Spearman rank correlation method. Protein origin time are categorized according to the data in OrthoMCL database. For (A)–(E), I, <1 Gya; II, 1–1.58 Gya; III, 1.58–1.84 Gya; IV, 1.84–2.23 Gya; V, 2.23–4 Gya; VI, >4 Gya. For (F), I, <2.6 Gya; II, 2.6–4 Gya; III, >4 Gya. *R* represents Spearman rank correlation coefficient and *P* represents its *P*-value. The values of upper and lower quartile are indicated as upper and lower edges of the box, and the values of median are indicated as a red bar in the box. The maximum whisker length is set as 1.5, which means points are drawn as outliers (dotted individually outside the bars) if they are larger than q3+1.5×(q3−q1) (shown as the upper bar) or smaller than q1−1.5×(q3−q1) (shown as the lower bar), where q1 and q3 are the 25th and 75th percentiles respectively. (***SCIN***: Spectral Count Index Normalized (11); ***SCI***: Spectral Count Index (11); ***NSAF***: Normalized Spectral Abundance Factor (12)).

An important evolutionary property of a protein is its origination time, which reflects its evolutionary span. Taking a proteome as a whole, we found that the abundance of proteins is positively correlated with the protein's origination time, though sometimes the correlations are not so strong (**Fig. 1** and **Table S1**). That is, the earlier a protein is born, the higher its abundance tends to be. This tendency exists in all the six species examined, indicating that it might be a general rule of organism phylogeny. One hypothesis is that the proteins that appeared first are likely to carry out essential housekeeping roles and are, therefore, required in higher abundance, whereas proteins that evolved later tend to act in specialized functions that do not need to be in high abundance. Enrichment analysis of the functional categories in proteins supports the hypothesis that proteins of housekeeping function or regulatory functions are clustered with distinct origination time in all the six species (**Table S2**).

**Figure 2:**
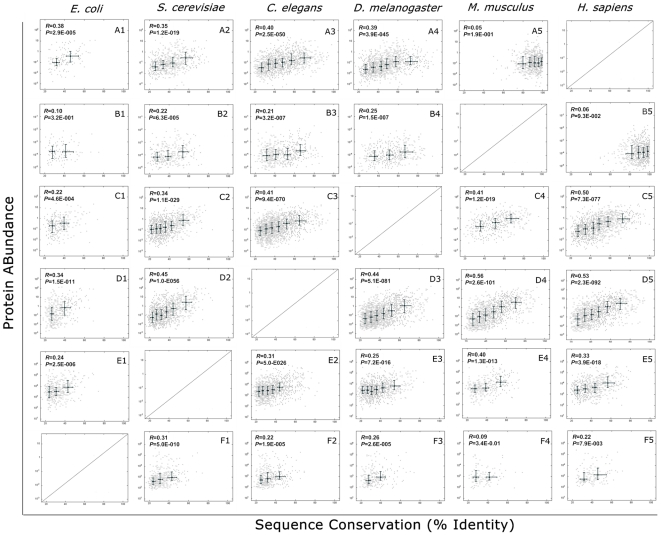
Proteome-wide correlation of proteins' abundance with their sequence conservation across six species. The abundance of a given *H. sapiens* protein was plotted with its orthologue sequence similarity: *H. sapiens vs. E. coli* (**A1**), *S. cerevisiae* (**A2**), *C. elegans* (**A3**), *D. melanogaster* (**A4**), and *M. musculus* (**A5**), respectively. The next five rows are for *M. musculus* (**B1–5**), *D. melanogaster* (**C1–5**), *C. elegans* (**D1–5**), *S. cerevisiae* (**E1–5**), and *E. coli* (**F1–5**), respectively. Medians of equal-sized bins are indicated as crosses; whiskers encompass the range from 25% to 75% of values. The orthologs of every two species are indicated as dots in the background. The correlation coefficients (*R*) between sequence conservation and abundance are shown in the inset.

An interesting question in molecular evolution is why proteins evolve at different rates [4]. A negative correlation between transcript abundance and the rate of sequence evolution, in which highly expressed (transcripts level) genes evolve slowly, has been shown to hold true across the species [6], while a positive correlation between protein abundance and sequence conservation has been recently discovered from the quantitative proteomic data of *C. elegans* and *D. Melanogaster* [30]. To determine whether this phenomenon can be extended towards two evolutionary directions beyond invertebrates, we investigated mammals and single-cell organisms–species that lie in two evolutionary directions from invertebrates–and indeed found a positive correlation between abundance and sequence conservation on the proteome level among most species examined (**Fig. 2**). Interestingly, we found that the correlation between protein conservation degree (human *vs.* mouse) and protein abundance is not significant (Fig. 2, A5, B5), which was never reported before. As for comparison of human and mouse, the two species showed high conservation and the identity of most proteins is over 80%, which lead to no relationship with abundance distribution. What's more, as for *E. coli*, the statistic data was not powerful in case that the homologous proteins were too little in the OrthoMCL database, which may partly lead to the low correlation between conservation degree (*E. coli vs.* mouse) and protein abundance (Fig. 2, B1 and F4).

### 2. Structural rule in protein abundance: domain versus abundance

The function of a protein is determined by its structure, which is mostly embodied in its domain architecture. We investigated the relationship between protein abundance and protein domain characteristics, including *DN* [31], [32] and *DC* [33]. *DN* is the number of annotated domains in the sequence of a protein, whereas *DC* is the percentage of the amino acid sequence that defines the domain over the whole protein sequence. As additional evidence, the number or coverage of domains mediating PPI (*PPI_DN* or *PPI_DC*) [33] are also employed in the analyses.

**Figure 3:**
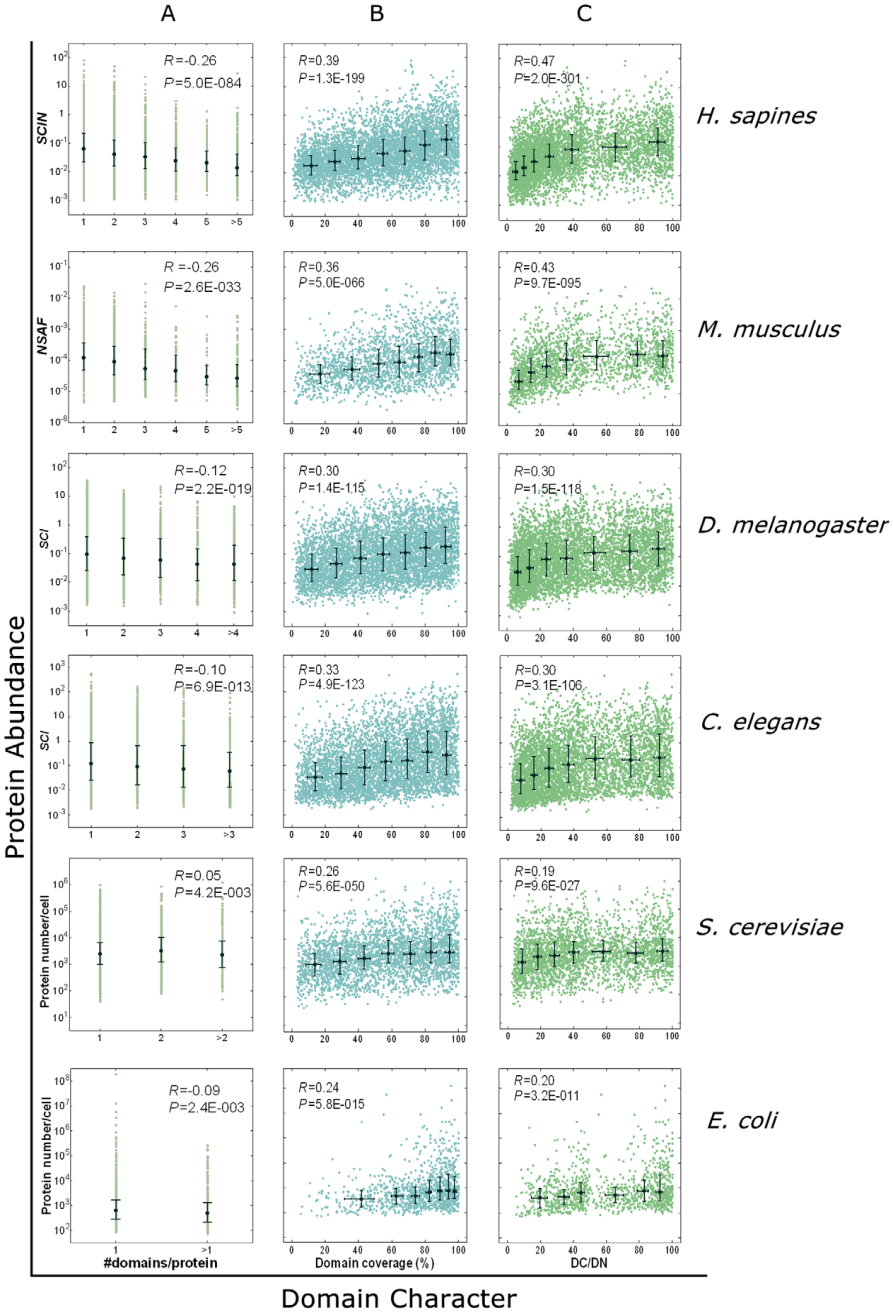
Proteome-wide correlation between proteins' abundance and their domain characters. Three parameters, namely, domain number (*DN*) (**A**), domain coverage (*DC*) (**B**), and *DC*/*DN* (**C**), were employed in the analyses. *R* represents Spearman rank correlation coefficient and *P* represents its *P*-value. Medians are indicated as black dots (**A**) or crosses (**B**, **C**), and whiskers encompass the range from 25% to 75% of values.

We found that the *DN* or *PPI_DN* of a protein is negatively correlated with its abundance, especially in higher eukaryotes (**Fig. 3A** and **Fig. S2A**). Notably, the correlations are not strong in the proteome data of *S. cerevisiae* and *E. coli*. This exception may be partly due to the fact that the proportions of multi-domain proteins in these two single-cellular organisms are much lower than other multi-cellular organisms, which is revealed by our analysis based on the data used in this study (**Fig. S3A**) and previous related studies [32]. We found the proportions of multi-domain proteins correlate well with the strengths of the correlations between *DN* and protein abundance (*R* = 0.9252, *P* = 0.0028, see **Fig. S3B**). In contrast, the *DC* or *PPI_DC* of proteins positively correlated with their abundance in the proteomes of all the six species (**Fig. 3B** and **Fig. S2B**).

*DN* can be regarded as the simplest parameter representing the complexity of a protein structure [31], [32], [34], and *PPI_DN* represents the complexity of the protein structure related to PPI. The smaller the *DN* or *PPI_DN*, the simpler the protein is. It can be inferred that proteins with simpler structures tend to be of higher abundance. Similarly, *DC* is an index of the structural compactness of a protein, and *PPI_DC* is an indicator of the structural compactness of PPI [33]. The significantly positive correlations between protein abundance and the proteins' *DC* or *PPI_DC* reveal that the more compact proteins are of higher abundance. To represent the integrated structural character, including complexity and compactness, we introduced a combined parameter, *DC*/*DN* (or *PPI_DC*/*PPI_DN*). We found that the *DC*/*DN* or *PPI_DC*/*PPI_DN* of proteins positively correlated with their abundance in the proteomes of all the six species (**Fig. 3C** and **Fig. S2C**). Thus, simple and compact proteins tend to exhibit high abundance, which does have some predictive power especially when proteins with vastly different domain characters are compared (**Table S3**).

Interestingly, the strength of correlation coefficient increases through the course of evolution (**Fig. 3** and **Fig. S2**), which may be related to the increasing of biological complexity. The abundance of proteins in the higher multi-cellular organisms may need more precise regulation, to meet with the complex functional requirement. This may lead to the stronger correlation between protein abundance and its complexity or compactness.

### 3. Functional rule in protein abundance: function versus abundance

**Figure 4:**
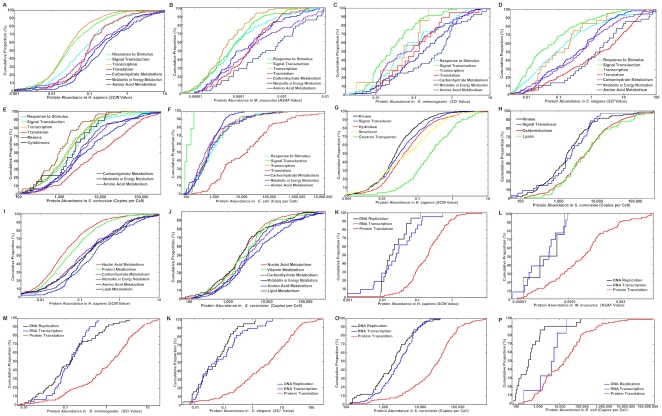
Proteome-wide correlation of proteins' abundance with their functional categorization across six species. Abundance distribution of proteins in the mass and the information categories was compared by cumulative curves in *H. sapiens* (**A**), *M. musculus* (**B**), *D. melanogaster* (**C**), *C. elegans* (**D**), *S. cerevisiae* (**E**), and *E. coli* (**F**). Stratified comparison: mass *vs.* information processing activities in *H. sapiens* (**G**) and *S. cerevisiae* (**H**); metabolism subclasses in *H. sapiens* (**I**) and *S. cerevisiae* (**J**). Comparison among biogenesis machines of three bio-molecules in *H. sapiens* (**K**), *M. musculus* (**L**), *D. melanogaster* (M), *C. elegans* (**N**), *S. cerevisiae* (**O**), and *E. coli* (**P**).

Cellular functions can be grouped into two categories: “mass category” and “information category”. Proteins in the mass category act on material conversion and transportation, including carbohydrate/lipid metabolism, amino acid/nucleotide metabolism, and energy metabolism; proteins in the information category act on information modulation and transduction, including response from extracellular stimulus to intracellular signal transduction, to gene regulation and expression. We found that from *H. sapiens* to *E. coli*, the abundance of proteins in the mass category is significantly higher than that of proteins in the information category (**Fig. 4A–F** and **Tables S4, S5**). For example, meiosis and cytokinesis proteins, which participate in a typical information process of *S. cerevisiae* that instantly decide the fate of a cell, have low abundance distributions (**Fig. 4E**). Many proteins in processing information have lower abundance than those in mass activities (**Fig. 4G, 4H**). Even in the metabolism category, which belongs to the mass category, nucleic acid and protein metabolisms, which are closer to information processing, exhibit lower abundance distributions than proteins involved in metabolisms of simpler compounds, such as lipid, amino acid, and carbohydrates (**Fig. 4I, 4J**).

The distinction of mass category and information category in protein abundance correlation is predicted by the evolutionary and structural rules. For example, human proteins in the mass category are highly enriched in proteins originating earlier than 4 Gya, and tend to be with lower *DN*, and higher *DC* and *PPI_DC* than those in the information category (**Fig. S4, B1–D6**). As expected from the Functional Rule in Protein Abundance, the central dogma of genetic information flow (DNA→mRNA→proteins) follows the trend of protein abundance up-regulation correlated with top/down information flow, in which proteins participating in DNA replication are of the lowest abundance, those in RNA transcription are of median abundance, and those in protein translation are of the highest abundance across species (**Fig. 4K–P** and **Table S6**). Also as predicted, along the biological information flow of “signalling→transcription→translation→metabolism”, downstream systems with higher abundance are of an earlier origination tendency than upstream ones (**Fig. S4, A1–6**).

## Discussion

Genome sequencing has enabled us to acquire a vast amount of data, and this has accelerated the discovery of biological rules at the genome level [35], [36]. In comparison with the genome and also transcriptome, the proteome is more dynamic and diverse in composition, modification, interaction, and localization. There remain many fundamental rules or regular patterns to be discovered that may explain the diverse nature of the proteome. With the advancement of proteomics, more and more studies began to mine the rules behind the huge datasets [37].

Protein abundance is one of important phenotypic variables of proteins, and is controlled precisely. Exploration for the proteome-wide relationship between the intrinsic properties (such as evolutionary, structural or functional characters) and this phenotypic variable will benefit to discovering the essential rules related to proteins. Here, we described three correlations of protein abundance with the protein's intrinsic properties of evolution, structure, and function, observed to be consistent from bacteria to yeast, worm, fly, mouse, and human: (1) There is a positive correlation with both the protein's origination time and sequence conservation during phylogeny, confirming the conclusions of previous studies at a wider range of species [4]–[6]; (2) We found protein abundance negatively correlated with the protein's domain number, and positively correlated with protein's domain coverage, which indicates that proteins with simple/compact structures tend to be high abundant. The finding that proteins with more domains tend to be with lower abundance may be due to the selection to reduce the risk of mis-translation [6] of the multi-domain proteins. Another phenomenon that higher abundant proteins tend to have higher domain coverage may result from the selection to reduce the length of non-functional regions within the abundant proteins to minimize transcriptional and translational costs, just as the similar selection on the length of intronic DNA [7]. (3) The abundances of proteins involved in the mass category tend to be higher than those in the information category, which also is similar to previous studies' conclusions obtained from the data of bacteria [11] or human cell line [14]. Our work firstly confirmed that this finding is presented across species, and some new findings emerged based on our new analyses. Intriguingly, the third pattern can be inferred from the first two patterns, highlighting the importance of the structure and function relationship.

Taken together, protein abundance distribution across the whole proteome displays prominent regularities, even though there are large discrepancies in protein composition and abundance among various proteomes of diverse bio-entities. Such regularities seem to be maintained in different organs as analysis of the mouse kidney dataset yielded identical patterns with the analysis of the mouse liver datasets (**Fig. S5**). The discovery of those regularities strongly demonstrated that the quantitative and comprehensive proteomic datasets could provide a rich ground for the exploration and discovery of the fundamental rules in nature on the proteome scale.

## Supporting Information

Figure S1 **Diversity time of all 87 species in OrthoMCL v2.** (**A**) Detailed diversity time tree that was constructed according to the reference (Hedges 2002). (**B**) Simplified diversity time tree with 7 significant time nodes to keep proper time scale distances (0.58 or 0.37, 0.26, 0.39, 1.77 and 1.40 Gya respectively) for analysis. The 6 species analyzed were in red text.
(PNG)

Figure S2 **Proteome-wide correlation between protein abundance and its protein-protein interacting (PPI) domain parameters.** Three Parameters including PPI domain number (*PPI_DN*) (**A**), PPI domain coverage (*PPI_DC*) (**B**) and *PPI_DC*/PPI_*DN* (**C**) were employed for the analyses. *R* represents Spearman rank correlation coefficient and *P* represents the *P*-value of the spearman rank correlation analysis. Medians are indicated as black dots (**A**), or crosses (**B**, **C**), and whiskers encompass the range from 25% to 75% of values.
(TIF)

Figure S3 **The proportion of multi-domain proteins in each dataset (A) and its relationship with the correlation coefficient values between domain number and protein abundance (B).** *E. coli* (Ec), *S. cerevisiae* (Sc), *C. elegans* (Ce), *D. melanogaster* (Dm), *M. musculus* cortex of kidney (MmC), *M. musculus* liver (MmL),and *H. sapiens* (Hs). *R* represents the pearson correlation coefficient, and *P* represent the *P*-value.
(PNG)

Figure S4 **Comparison of mass categorial proteins with information categorial ones on distributions of origin time and domain character among six species.** Origin time distributions of proteins in *H. sapiens* (**a1**), *M. musculus* (**a2**), *D. melanogaster* (**a3**), *C. elegans* (**a4**), *S. cerevisiae* (**a5**) and *E. coli* (**a6**). Domain character distributions of proteins in the same species: *DN* (**b1–6**), *DC* (**c1–6**) and *PPI_DC* (**d1–6**).
(PNG)

Figure S5 **Consistency of the three rules across tissues.** When replacing mouse kidney data by mouse liver data in analyses, all rules were maintained. These analyses include: correlations of protein abundance with origin time (**A**), sequence conservation (**B**) domain characters (**C**), functional categories (**D**) and biogenesis machines of three bio-molecules (**E**).
(TIF)

Table S1 **Rank sum test** ***p*** **-values between origin time categorized proteins' abundance datasets.**
(DOC)

Table S2 **The functional enrichment results of different origin time protein categories in 6 species by DAVID.**
(DOC)

Table S3 **Predictive power of each domain character parameter correlating with protein abundance.**
(DOC)

Table S4 **Rank sum test p-values between various functional categorized proteins' abundance datasets across six species.**
(DOC)

Table S5 **Rank sum test** ***p*** **-values between various functional categorized proteins' abundance** ***datasets in H. sapiens*** **and** ***S. cerevisiae*** **.**
(DOC)

Table S6 **Rank sum test p-values between proteins' abundance datasets of three bio-molecules biogenesis machines across six species.**
(DOC)
