# Supplementary figures and images for: Regular Patterns for Proteome-Wide Distribution of Protein Abundance across Species
Source: PLoS One. 2012 Mar 9;7(3):e32423. doi: 10.1371/journal.pone.0032423 (PMC3302874; doi:10.1371/journal.pone.0032423)

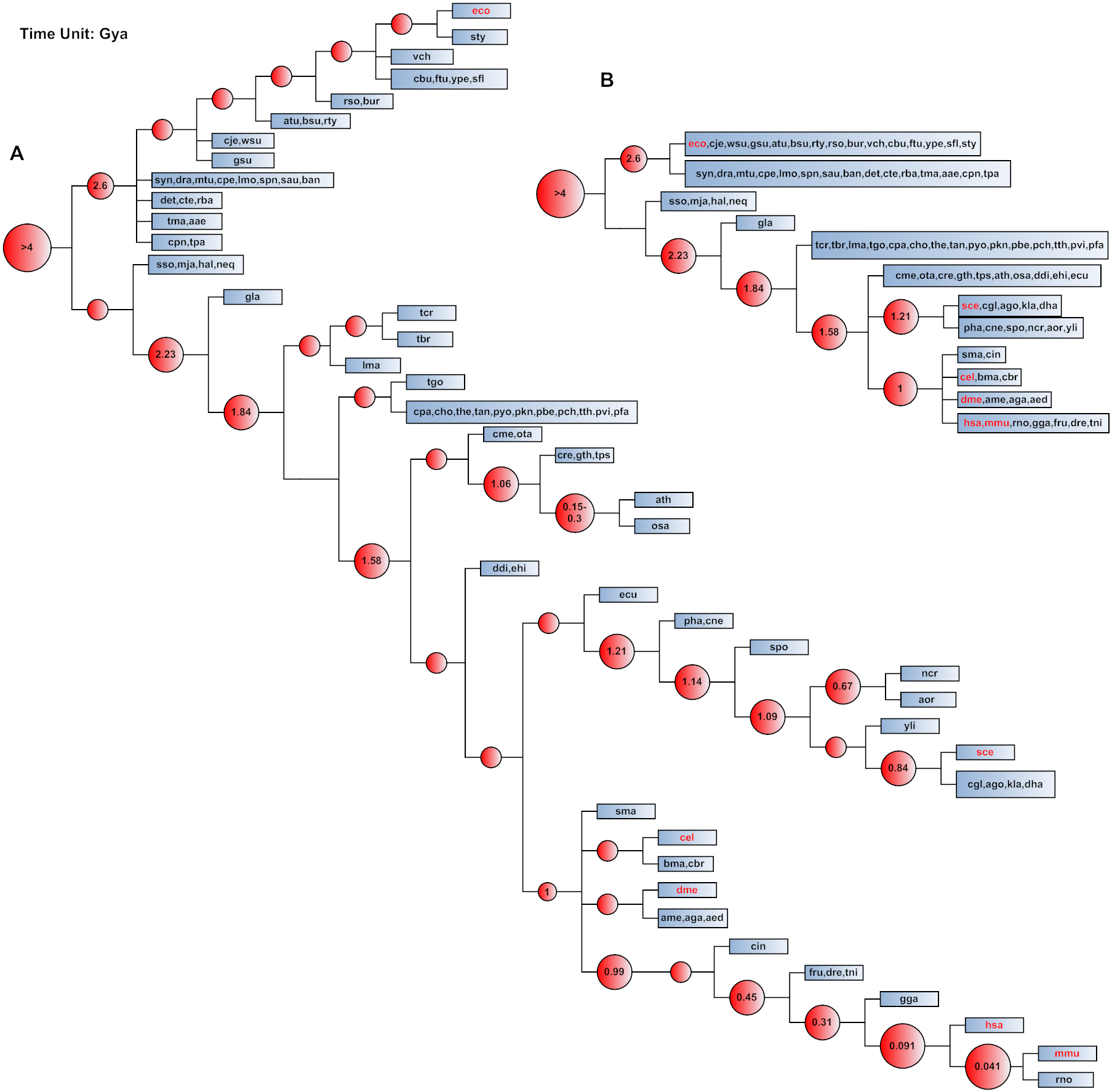

Supplement: Figure S1 — Diversity time of all 87 species in OrthoMCL v2. (A) Detailed diversity time tree that was constructed according to the reference (Hedges 2002). (B) Simplified diversity time tree with 7 significant time nodes to keep proper time scale distances (0.58 or 0.37, 0.26, 0.39, 1.77 and 1.40 Gya respectively) for analysis. The 6 species analyzed were in red text. (PNG) [file pone.0032423.s001.png]

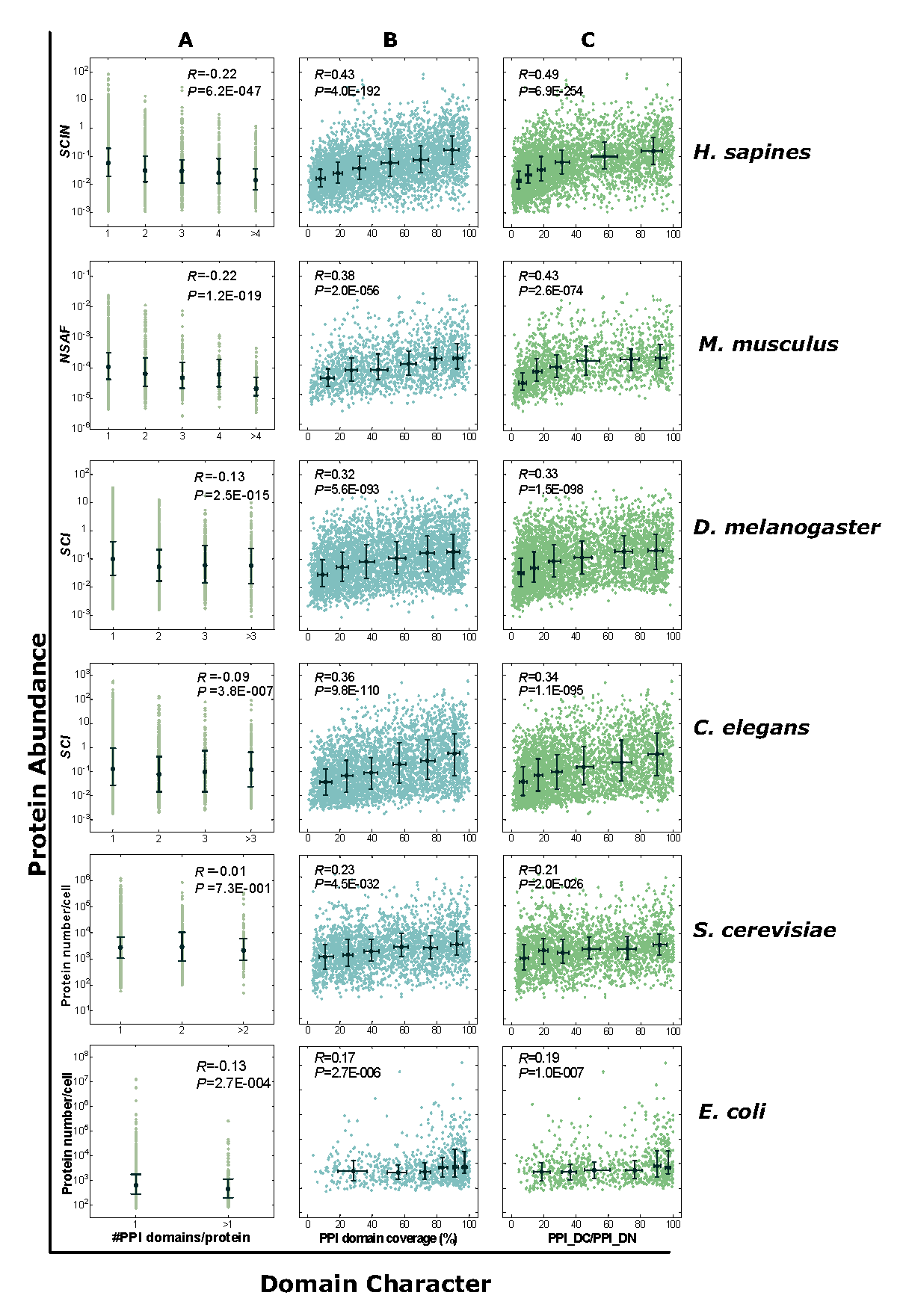

Supplement: Figure S2 — Proteome-wide correlation between protein abundance and its protein-protein interacting (PPI) domain parameters. Three Parameters including PPI domain number (PPI_DN) (A), PPI domain coverage (PPI_DC) (B) and PPI_DC/PPI_DN (C) were employed for the analyses. R represents Spearman rank correlation coefficient and P represents the P-value of the spearman rank correlation analysis. Medians are indicated as black dots (A), or crosses (B, C), and whiskers encompass the range from 25% to 75% of values. (TIF) [file pone.0032423.s002.tif]

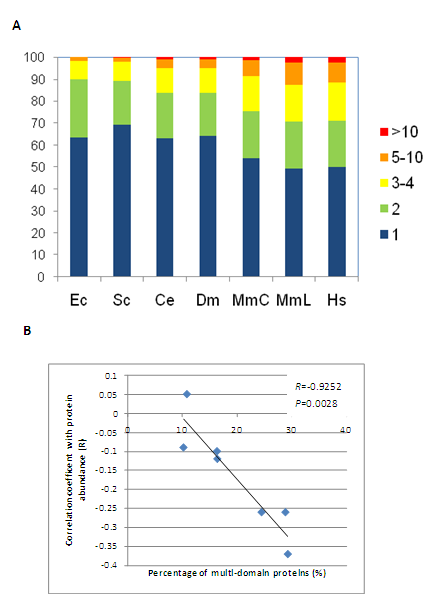

Supplement: Figure S3 — The proportion of multi-domain proteins in each dataset (A) and its relationship with the correlation coefficient values between domain number and protein abundance (B). E. coli (Ec), S. cerevisiae (Sc), C. elegans (Ce), D. melanogaster (Dm), M. musculus cortex of kidney (MmC), M. musculus liver (MmL),and H. sapiens (Hs). R represents the pearson correlation coefficient, and P represent the P-value. (PNG) [file pone.0032423.s003.png]

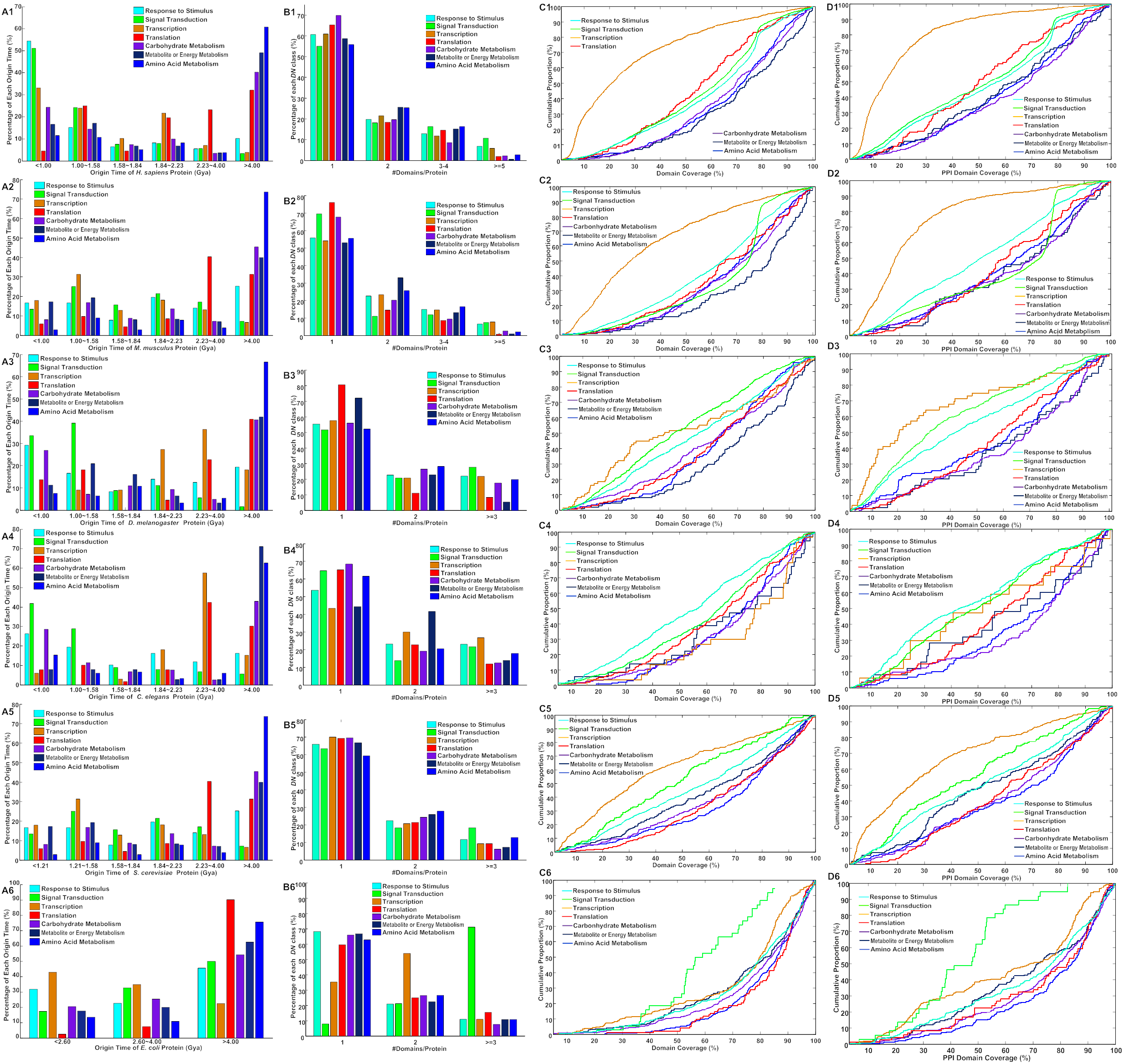

Supplement: Figure S4 — Comparison of mass categorial proteins with information categorial ones on distributions of origin time and domain character among six species. Origin time distributions of proteins in H. sapiens (a1), M. musculus (a2), D. melanogaster (a3), C. elegans (a4), S. cerevisiae (a5) and E. coli (a6). Domain character distributions of proteins in the same species: DN (b1–6), DC (c1–6) and PPI_DC (d1–6). (PNG) [file pone.0032423.s004.png]

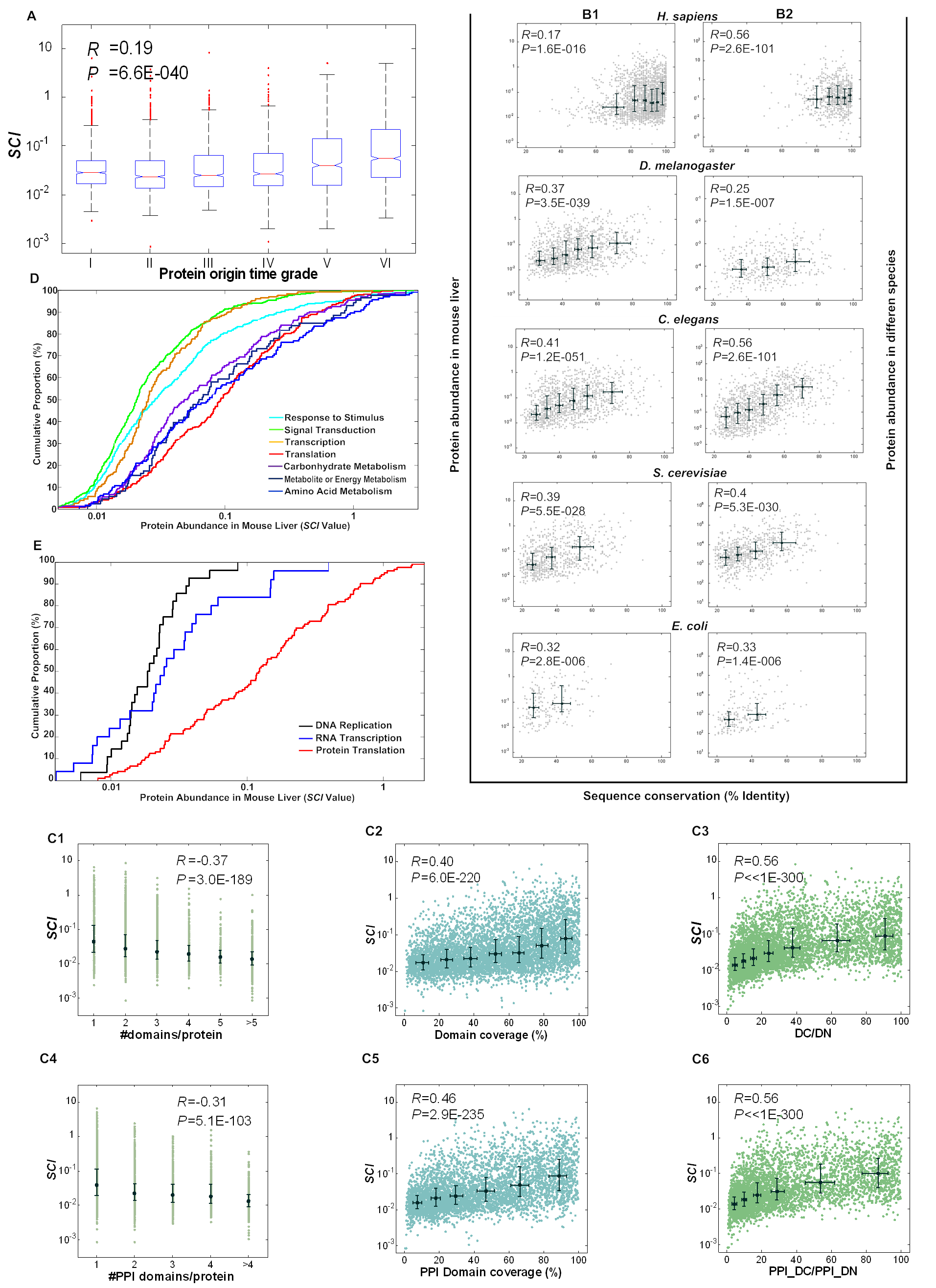

Supplement: Figure S5 — Consistency of the three rules across tissues. When replacing mouse kidney data by mouse liver data in analyses, all rules were maintained. These analyses include: correlations of protein abundance with origin time (A), sequence conservation (B) domain characters (C), functional categories (D) and biogenesis machines of three bio-molecules (E). (TIF) [file pone.0032423.s005.tif]
