# Supplementary material for: Regular Patterns for Proteome-Wide Distribution of Protein Abundance across Species
Source: PLoS One. 2012 Mar 9;7(3):e32423. doi: 10.1371/journal.pone.0032423 (PMC3302874; doi:10.1371/journal.pone.0032423)
Supplement: Table S1 — Rank sum test p -values between origin time categorized proteins' abundance datasets. (DOC) [file pone.0032423.s006.doc]

| **Supplementary Table 1. Rank sum test *p*-values between origin time categorized proteins’ abundance datasets.** |
| --- |
| | <1.00 Gya | <1.00 Gya |  |  | *H. sapiens* (Liver) | | | --- | --- | --- | --- | --- | --- | | 1.00~1.58 Gya | 0.0316 | 1.00~1.58 Gya |  | | 1.58~1.84 Gya | 0.4405 | 0.3557 | 1.58~1.84 Gya |  |  | | 1.84~2.23 Gya | 0.6618 | 0.0089 | 0.2443 | 1.84~2.23 Gya |  | | 2.23~4.00 Gya | 6.6019×10^-6^ | 3.9039×10^-10^ | 3.4190×10^-6^ | 2.0242×10^-5^ | 2.23~4.00 Gya | | >4.00 Gya | 1.3050×10^-22^ | 2.7880×10^-32^ | 3.5180×10^-20^ | 4.4276×10^-23^ | 6.4836×10^-6^ | |
| | <1.00 Gya | <1.00 Gya |  |  | *M. musculus* (Renal cortex) | | | --- | --- | --- | --- | --- | --- | | 1.00~1.58 Gya | 0.0905 | 1.00~1.58 Gya |  | | 1.58~1.84 Gya | 0.6213 | 0.0393 | 1.58~1.84 Gya |  |  | | 1.84~2.23 Gya | 0.6242 | 0.1527 | 0.2703 | 1.84~2.23 Gya |  | | 2.23~4.00 Gya | 0.0016 | 3.2565×10^-7^ | 0.0146 | 2.0305×10^-5^ | 2.23~4.00 Gya | | >4.00 Gya | 4.5169×10^-8^ | 2.7613×10^-15^ | 9.6832×10^-7^ | 1.3890×10^-13^ | 0.0054 | |
| | <1.00 Gya | <1.00 Gya |  |  | *M. musculus* (Liver) | | | --- | --- | --- | --- | --- | --- | | 1.00~1.58 Gya | 1.4659×10^-4^ | 1.00~1.58 Gya |  | | 1.58~1.84 Gya | 0.1750 | 0.0977 | 1.58~1.84 Gya |  |  | | 1.84~2.23 Gya | 0.9874 | 9.5387×10^-4^ | 0.2698 | 1.84~2.23 Gya |  | | 2.23~4.00 Gya | 4.6242×10^-7^ | 4.4111×10^-13^ | 3.1737×10^-6^ | 1.9757×10^-5^ | 2.23~4.00 Gya | | >4.00 Gya | 3.1254×10^-34^ | 8.9230×10^-47^ | 2.4582×10^-24^ | 2.4548×10^-26^ | 1.4056×10^-6^ | |
| | <1.00 Gya | <1.00 Gya |  |  | *D. melanogaster* | | | --- | --- | --- | --- | --- | --- | | 1.00~1.58 Gya | 0.3734 | 1.00~1.58 Gya |  | | 1.58~1.84 Gya | 4.1603×10^-6^ | 4.0548×10^-4^ | 1.58~1.84 Gya |  |  | | 1.84~2.23 Gya | 1.4249×10^-25^ | 1.6549×10^-20^ | 6.2335×10^-7^ | 1.84~2.23 Gya |  | | 2.23~4.00 Gya | 1.0179×10^-49^ | 2.6389×10^-45^ | 8.7714×10^-27^ | 2.2791×10^-14^ | 2.23~4.00 Gya | | >4.00 Gya | 3.2014×10^-40^ | 1.3495×10^-35^ | 1.4634×10^-16^ | 1.6495×10^-5^ | 3.4569×10^-4^ | |
| | <1.00 Gya | <1.00 Gya |  |  | *C. elegans* | | | --- | --- | --- | --- | --- | --- | | 1.00~1.58 Gya | 0.9155 | 1.00~1.58 Gya |  | | 1.58~1.84 Gya | 0.6543 | 0.7815 | 1.58~1.84 Gya |  |  | | 1.84~2.23 Gya | 2.5047×10^-4^ | 0.0061 | 0.0321 | 1.84~2.23 Gya |  | | 2.23~4.00 Gya | 3.2318×10^-52^ | 5.4992×10^-34^ | 2.0437×10^-30^ | 2.9647×10^-31^ | 2.23~4.00 Gya | | >4.00 Gya | 1.5074×10^-49^ | 3.9685×10^-28^ | 3.3125×10^-24^ | 8.6281×10^-24^ | 2.2521×10^-4^ | |
| | <1.21 Gya | <1.21 Gya |  |  | *S. cerevisiae* | | | --- | --- | --- | --- | --- | --- | | 1.21~1.58 Gya | 0.0101 | 1.21~1.58 Gya |  | | 1.58~1.84 Gya | 4.3123×10^-9^ | 1.6946×10^-4^ | 1.58~1.84 Gya |  |  | | 1.84~2.23 Gya | 4.4792×10^-12^ | 3.7149×10^-6^ | 0.9589 | 1.84~2.23 Gya |  | | 2.23~4.00 Gya | 4.5218×10^-49^ | 1.9482×10^-41^ | 1.4451×10^-15^ | 3.4722×10^-22^ | 2.23~4.00 Gya | | >4.00 Gya | 1.4159×10^-35^ | 4.4116×10^-27^ | 1.5614×10^-7^ | 4.9056×10^-11^ | 4.9019×10^-4^ | |
| | <2.60 Gya | <2.60 Gya | *E. coli* | | --- | --- | --- | | 2.60~4.00 Gya | 0.7674 | 2.60~4.00 Gya | | >4.00 Gya | 2.0570×10^-4^ | 0.0017 | |
