# Supplementary material for: Regular Patterns for Proteome-Wide Distribution of Protein Abundance across Species
Source: PLoS One. 2012 Mar 9;7(3):e32423. doi: 10.1371/journal.pone.0032423 (PMC3302874; doi:10.1371/journal.pone.0032423)
Supplement: Table S2 — The functional enrichment results of different origin time protein categories in 6 species by DAVID. (DOC) [file pone.0032423.s007.doc]

Supplementary Table 2. The functional enrichment results of different origin time protein categories in 6 species by DAVID.

| **Origin Time (Gya)** | **>4.00** | **2.23～4.00** | **1.84～2.23** | **1.58～1.84** | **1.00～1.58** | **<1.00** |
| --- | --- | --- | --- | --- | --- | --- |
| ***H. sapiens*** | Transit peptide**  ATP binding**  Cofactor metabolism** | Translation**  ATP binding*  Protein catabolism* | mRNA processing*  Protein transport  Protein catabolism | GTPase regulator | Pleckstrin homology  Cytoskeleton  PDZ domain | Cytoskeletal keratin*  Heparin binding  Response to wounding |
| ***M. musculus*** | Transit peptide**  Carbohydrate metabolism**  NAD* | Translation**  ATP binding*  Protein transport | mRNA processing*  Protein transport*  Translation | Actin cytoskeleton  Protein transport | Transit peptide  Src homology-3  Actin binding |  |
| ***D. melanogaster*** | ATP binding**  NAD(P)-binding domain**  Transit peptide** | ATP binding**  Cell cycle**  Translation* | mRNA processing*  Protein transport*  Protein catabolism |  | Neuron*  Pleckstrin homology  Cell adhesion | Cuticle**  CHK kinase*  Chymotrypsin |
| ***C. elegans*** | Transit peptide**  ATP binding**  Flavoprotein** | Larval development**  Translation**  Growth** | Larval development**  Reproduction**  RNA recognition motif* |  | Pleckstrin homology*  Src homology-3*  Cell adhesion | Cuticle collagen**  Cuticle |
| **Origin Time (Gya)** | **>4.00** | **2.23～4.00** | **1.84～2.23** | **1.58～1.84** | **1.21～1.58** | **<1.21** |
| ***S. cerevisiae*** | AA. metabolism**  Transit peptide**  Cofactor metabolism** | Translation**  rRNA processing**  Translation regulation** | mRNA processing*  Protein transport  Protein catabolism | Transcription | Transcription*  Transcription factor |  |
| **Origin Time (Gya)** | **>4.00** | **2.60～4.00** | **<2.60** | | | |
| ***E. coli*** | Nucleotide binding**  Oxidoreductase**  Metal ion binding** |  | Signalling* | | | |
| Each category lists the top 3 enrichment items with enrichment score ≥5. *Enrichment score ≥10; **Enrichment score ≥20. AA.: amino acid. | | | | | | |
