# Supplementary material for: Regular Patterns for Proteome-Wide Distribution of Protein Abundance across Species
Source: PLoS One. 2012 Mar 9;7(3):e32423. doi: 10.1371/journal.pone.0032423 (PMC3302874; doi:10.1371/journal.pone.0032423)
Supplement: Table S3 — Predictive power of each domain character parameter correlating with protein abundance. (DOC) [file pone.0032423.s008.doc]

**Supplementary Table 3. Predictive power of each domain character parameter correlating with protein abundance.**

| Data  source | Domain characters *vs.* Protein abundance (predictive power) | | | | | | | | | | | |
| --- | --- | --- | --- | --- | --- | --- | --- | --- | --- | --- | --- | --- |
|  | *DN* | | *DC* | | *DC*/*DN* | | *PPI_DN* | | *PPI_DC* | | *PPI_DC*/*PPI_DN* | |
|  | Min* | Max* | Min | Max | Min | Max | Min | Max | Min | Max | Min | Max |
| HLP | 61.65 | 73.80 | 63.20 | 84.38 | 66.11 | 88.88 | 61.31 | 73.00 | 64.72 | 89.83 | 66.81 | 91.44 |
| MCP | 62.22 | 82.02 | 62.20 | 87.70 | 64.63 | 89.24 | 62.25 | 82.07 | 62.76 | 90.00 | 64.60 | 90.50 |
| MLP | 66.73 | 85.52 | 63.35 | 87.58 | 69.17 | 91.87 | 66.22 | 81.60 | 65.69 | 92.04 | 69.49 | 94.58 |
| Fly | 56.33 | 65.11 | 60.07 | 83.30 | 60.22 | 83.15 | 57.67 | 58.42 | 60.73 | 84.91 | 61.06 | 84.87 |
| Worm | 55.26 | 58.24 | 60.93 | 77.86 | 60.20 | 75.98 | 55.19 | 56.01 | 62.20 | 82.99 | 61.42 | 80.14 |
| Yeast | 46.95 | 49.61 | 58.79 | 73.35 | 56.30 | 73.82 | 50.36 | 53.91 | 57.75 | 76.82 | 57.00 | 75.74 |
| *E. coli* | 54.87 | 55.59 | 57.64 | 74.62 | 56.51 | 71.41 | 57.89 | 62.53 | 55.22 | 60.95 | 56.02 | 64.41 |

* The “Min” and “Max” values are obtained when selected various subsets of protein pairs with a minimum rank difference percentage from 0 to 95% (see method section for detail).
