# Supplementary material for: Regular Patterns for Proteome-Wide Distribution of Protein Abundance across Species
Source: PLoS One. 2012 Mar 9;7(3):e32423. doi: 10.1371/journal.pone.0032423 (PMC3302874; doi:10.1371/journal.pone.0032423)
Supplement: Table S4 — Rank sum test p-values between various functional categorized proteins' abundance datasets across six species. (DOC) [file pone.0032423.s009.doc]

**Supplementary Table 4. Rank sum test *p*-values between various functional categorized proteins’ abundance datasets across six species.**

| Response to  Stimulus | Response to  Stimulus |  |  | *H. sapiens* (Liver) | | |
| --- | --- | --- | --- | --- | --- | --- |
| Signal  Transduction | 1.3627×10^-14^ | Signal  Transduction |  |
| Transcription | 8.6899×10^-20^ | 0.0763 | Transcription |
| Translation | 0.0245 | 1.0983×10^-11^ | 5.1723×10^-16^ | Translation |  |  |
| Carbohydrate  Metabolism | 8.8976×10^-5^ | 1.0719×10^-19^ | 6.5699×10^-24^ | 0.3421 | Carbohydrate  Metabolism |  |
| Metabolite or  Energy Metabolism | 7.9695×10^-17^ | 1.6281×10^-45^ | 1.3306×10^-50^ | 1.3307×10^-4^ | 0.0066 | Metabolite or  Energy Metabolism |
| AA. Metabolism | 3.5641×10^-10^ | 4.7201×10^-29^ | 1.7518×10^-34^ | 0.0037 | 0.0321 | 0.6740 |

| Response to  Stimulus | Response to  Stimulus |  |  | *M. musculus* (Renal cortex) | | |
| --- | --- | --- | --- | --- | --- | --- |
| Signal  Transduction | 0.4337 | Signal  Transduction |  |
| Transcription | 0.0250 | 0.0908 | Transcription |
| Translation | 1.5265×10^-5^ | 1.9798×10^-8^ | 1.6638×10^-10^ | Translation |  |  |
| Carbohydrate  Metabolism | 2.0311×10^-4^ | 3.2379×10^-6^ | 1.8469×10^-7^ | 0.6147 | Carbohydrate  Metabolism |  |
| Metabolite or  Energy Metabolism | 1.2169×10^-6^ | 1.3066×10^-8^ | 1.2375×10^-9^ | 0.0030 | 0.0541 | Metabolite or  Energy Metabolism |
| AA. Metabolism | 0.0125 | 6.0159×10^-4^ | 1.8958×10^-5^ | 0.3508 | 0.2505 | 0.0024 |

| Response to  Stimulus | Response to  Stimulus |  |  | *M. musculus* (Liver) | | |
| --- | --- | --- | --- | --- | --- | --- |
| Signal  Transduction | 1.9218×10^-5^ | Signal  Transduction |  |
| Transcription | 0.2910 | 0.0020 | Transcription |
| Translation | 6.2621×10^-18^ | 1.8038×10^-40^ | 1.5379×10^-23^ | Translation |  |  |
| Carbohydrate  Metabolism | 5.4986×10^-7^ | 2.8319×10^-19^ | 3.9160×10^-10^ | 0.0042 | Carbohydrate  Metabolism |  |
| Metabolite or  Energy Metabolism | 9.2518×10^-7^ | 4.8921×10^-15^ | 7.0257×10^-10^ | 0.2557 | 0.2709 | Metabolite or  Energy Metabolism |
| AA. Metabolism | 6.4307×10^-9^ | 1.4276×10^-19^ | 9.7688×10^-12^ | 0.5321 | 0.1259 | 0.7300 |

| Response to  Stimulus | Response to  Stimulus |  |  | *D. melanogaster* | | |
| --- | --- | --- | --- | --- | --- | --- |
| Signal  Transduction | 0.0079 | Signal  Transduction |  |
| Transcription | 0.6679 | 0.2821 | Transcription |
| Translation | 0.5032 | 0.0036 | 0.3180 | Translation |  |  |
| Carbohydrate  Metabolism | 0.9336 | 0.0013 | 0.5750 | 0.4934 | Carbohydrate  Metabolism |  |
| Metabolite or  Energy Metabolism | 0.0070 | 1.5912×10^-7^ | 0.0237 | 0.0555 | 0.0022 | Metabolite or  Energy Metabolism |
| AA. Metabolism | 0.0277 | 7.1310×10^-8^ | 0.1030 | 0.3084 | 0.0099 | 0.1845 |

| Response to  Stimulus | Response to  Stimulus |  |  | *C. elegans* | | |
| --- | --- | --- | --- | --- | --- | --- |
| Signal  Transduction | 1.7676×10^-5^ | Signal  Transduction |  |
| Transcription | 0.5893 | 0.0058 | Transcription |
| Translation | 8.0175×10^-18^ | 5.3911×10^-27^ | 3.9228×10^-5^ | Translation |  |  |
| Carbohydrate  Metabolism | 6.7792×10^-7^ | 2.5391×10^-14^ | 0.0202 | 2.1247×10^-4^ | Carbohydrate  Metabolism |  |
| Metabolite or  Energy Metabolism | 7.0703×10^-7^ | 1.6611×10^-10^ | 2.8455×10^-4^ | 0.8412 | 0.0193 | Metabolite or  Energy Metabolism |
| AA. Metabolism | 2.0422×10^-5^ | 2.7123×10^-13^ | 0.0514 | 1.9169×10^-6^ | 0.4062 | 0.0025 |

| **Response to Stimulus** | **Response to Stimulus** |  |  |  | *S. cerevisiae* | | | |
| --- | --- | --- | --- | --- | --- | --- | --- | --- |
| **Signal Transduction** | **0.0278** | **Signal Transduction** |  |  |
| **Transcription** | **0.0049** | **0.7862** | **Transcription** |  |
| **Translation** | **1.1086×10^-27^** | **3.1269×10^-21^** | **2.1930×10^-36^** | **Translation** |
| **Meiosis** | **1.9589×10^-4^** | **0.0312** | **0.0092** | **2.9744×10^-16^** | **Meiosis** |  |  |  |
| **Cytokinesis** | **0.8842** | **0.3829** | **0.4400** | **4.2287×10^-5^** | **0.0359** | **Cytokinesis** |  |  |
| **Carbohydrate**  **Metabolism** | **1.2972×10^-5^** | **3.1019×10^-7^** | **1.2712×10^-10^** | **1.4775×10^-7^** | **1.5366×10^-8^** | **0.0673** | **Carbohydrate**  **Metabolism** |  |
| **Metabolite or**  **Energy Metabolism** | **7.2801×10^-4^** | **8.6075×10^-6^** | **4.3495×10^-8^** | **8.4875×10^-9^** | **1.0604×10^-7^** | **0.1411** | **0.4395** | **Metabolite or**  **Energy Metabolism** |
| **AA. Metabolism** | **1.6297×10^-9^** | **1.9251×10^-10^** | **1.2952×10^-15^** | **1.6654×10^-4^** | **1.2537×10^-10^** | **0.0106** | **0.1336** | **0.0319** |

| Response to  Stimulus | Response to  Stimulus |  |  | *E. coli* | | |
| --- | --- | --- | --- | --- | --- | --- |
| Signal  Transduction | 6.7873×10^-4^ | Signal  Transduction |  |
| Transcription | 0.3812 | 4.2946×10^-4^ | Transcription |
| Translation | 3.7314×10^-15^ | 1.8346×10^-4^ | 4.8102×10^-15^ | Translation |  |  |
| Carbohydrate  Metabolism | 0.0644 | 6.9960×10^-4^ | 0.3604 | 1.7590×10^-22^ | Carbohydrate  Metabolism |  |
| Metabolite or  Energy Metabolism | 0.6320 | 0.0015 | 0.7739 | 2.0942×10^-15^ | 0.2438 | Metabolite or  Energy Metabolism |
| AA. Metabolism | 0.1450 | 4.8485×10^-4^ | 0.8546 | 1.1460×10^-24^ | 0.2849 | 0.5442 |
