# Supplementary material for: Regular Patterns for Proteome-Wide Distribution of Protein Abundance across Species
Source: PLoS One. 2012 Mar 9;7(3):e32423. doi: 10.1371/journal.pone.0032423 (PMC3302874; doi:10.1371/journal.pone.0032423)
Supplement: Table S5 — Rank sum test p -values between various functional categorized proteins' abundance datasets in H. sapiens and S. cerevisiae . (DOC) [file pone.0032423.s010.doc]

**Supplementary Table 5. Rank sum test *p*-values between various functional categorized proteins’ abundance *datasets in H. sapiens* and *S. cerevisiae*.**

| Kinase | Kinase |  | *H. sapiens* | |
| --- | --- | --- | --- | --- |
| Signal Transducer | 0.0124 | Signal Transducer |
| Hydrolase | 1.7560×10^-8^ | 0.0010 | Hydrolase |  |
| Structural | 6.9299×10^-6^ | 0.0046 | 0.6346 | Structural |
| Electron Transporter | 1.2989×10^-27^ | 1.2923×10^-23^ | 1.3475×10^-19^ | 9.2567×10^-13^ |

| Kinase | Kinase |  | *S. cerevisiae* |
| --- | --- | --- | --- |
| Signal Transducer | 0.5859 | Signal Transducer |  |
| Oxidoreductase | 1.2320×10^-9^ | 0.0015 | Oxidoreductase |
| Lyase | 4.9339×10^-6^ | 0.0038 | 0.8199 |

| Nuclei Acid Metabolism | Nuclei Acid Metabolism |  |  | *H. sapiens* | |
| --- | --- | --- | --- | --- | --- |
| Protein Metabolism | 5.3071×10^-7^ | Protein Metabolism |  |
| Carbohydrate Metabolism | 8.5235×10^-15^ | 6.1036×10^-8^ | Carbohydrate Metabolism |  |  |
| Metabolite or Energy Metabolism | 7.1668×10^-40^ | 4.8015×10^-27^ | 0.0066 | Metabolite or Energy Metabolism |  |
| AA. Metabolism | 2.6027×10^-24^ | 2.0453×10^-15^ | 0.0321 | 0.6740 | AA. Metabolism |
| Lipid Metabolism | 4.3347×10^-19^ | 5.4969×10^-11^ | 0.5469 | 0.0366 | 0.1197 |

| Nuclei Acid Metabolism | Nuclei Acid Metabolism |  |  | *S. cerevisiae* | |
| --- | --- | --- | --- | --- | --- |
| Vitamin Metabolism | 0.4052 | Vitamin Metabolism |  |
| Carbohydrate Metabolism | 3.1859×10^-6^ | 0.1069 | Carbohydrate Metabolism |  |  |
| Metabolite or Energy Metabolism | 4.0505×10^-4^ | 0.2746 | 0.4395 | Metabolite or Energy Metabolism |  |
| AA. Metabolism | 8.5053×10^-11^ | 0.0093 | 0.1336 | 0.0319 | AA. Metabolism |
| Lipid Metabolism | 0.9503 | 0.4789 | 0.0012 | 0.0097 | 1.3080×10^-5^ |
