# Supplementary material for: Regular Patterns for Proteome-Wide Distribution of Protein Abundance across Species
Source: PLoS One. 2012 Mar 9;7(3):e32423. doi: 10.1371/journal.pone.0032423 (PMC3302874; doi:10.1371/journal.pone.0032423)
Supplement: Table S6 — Rank sum test p-values between proteins' abundance datasets of three bio-molecules biogenesis machines across six species. (DOC) [file pone.0032423.s011.doc]

**Supplementary Table 6. Rank sum test p-values between proteins’ abundance datasets of three bio-molecules biogenesis machines across six species.**

|  | DNA replication  *vs.*  RNA transcription | DNA replication  *vs.*  Protein translation | RNA transcription  *vs.*  Protein translation |
| --- | --- | --- | --- |
| *H. sapiens* (Liver) | 0.5297 | 3.8079×10^-8^ | 2.3082×10^-7^ |
| *M. musculus* (Renal cortex) | 0.9563 | 0.0270 | 6.0118×10^-5^ |
| *M. musculus* (Liver) | 0.2541 | 6.6646×10^-10^ | 6.1313×10^-6^ |
| *D. melanogaster* | 0.9454 | 2.9489×10^-11^ | 1.4028×10^-11^ |
| *C. elegans* | 0.2525 | 3.1685×10^-14^ | 8.9707×10^-12^ |
| *S. cerevisiae* | 0.0913 | 1.2210×10^-18^ | 1.8850×10^-23^ |
| *E. coli* | 0.0237 | 1.4145×10^-8^ | 0.1254 |
